# RETRACTION: Effect of Segmental Versus Lobectomy in Minimally Invasive Surgery on Postoperative Wound Complications in Lung Cancer Patients: A Meta‐Analysis

**DOI:** 10.1111/iwj.70366

**Published:** 2025-03-17

## Abstract

**RETRACTION**: ZhouJ. and WangW. , “Effect of Segmental Versus Lobectomy in Minimally Invasive Surgery on Postoperative Wound Complications in Lung Cancer Patients: A Meta‐Analysis,” International Wound Journal 21, no. 2 (2024): e14455, 10.1111/iwj.14455.PMC1082852537947029

The above article, published online on 10 November 2023, in Wiley Online Library (http://onlinelibrary.wiley.com/), has been retracted by agreement between the journal Editor in Chief, Professor Keith Harding; and John Wiley & Sons Ltd. Following an investigation by the publisher, all parties have concluded that this article was accepted solely on the basis of a compromised peer review process. The editors have therefore decided to retract the article. The authors did not respond to our notice regarding the retraction.

**RETRACTION**: J. Zhou and W. Wang , “Effect of Segmental Versus Lobectomy in Minimally Invasive Surgery on Postoperative Wound Complications in Lung Cancer Patients: A Meta‐Analysis,” International Wound Journal 21, no. 2 (2024): e14455, 10.1111/iwj.14455.PMC1082852537947029

The above article, published online on 10 November 2023, in Wiley Online Library (http://onlinelibrary.wiley.com/), has been retracted by agreement between the journal Editor in Chief, Professor Keith Harding; and John Wiley & Sons Ltd. Following an investigation by the publisher, all parties have concluded that this article was accepted solely on the basis of a compromised peer review process. The editors have therefore decided to retract the article. The authors did not respond to our notice regarding the retraction.

